# Isolate sets partition benefits community detection of parallel Louvain method

**DOI:** 10.1038/s41598-022-11987-y

**Published:** 2022-05-17

**Authors:** Hang Qie, Shijie Li, Yong Dou, Jinwei Xu, Yunsheng Xiong, Zikai Gao

**Affiliations:** School of Computer, National University of Defense Technology, Changsha, 410073 China

**Keywords:** Computer science, Information technology, Scientific data

## Abstract

Community detection is a vital task in many fields, such as social networks, and financial analysis, to name a few. The Louvain method, the main workhorse of community detection, is a popular heuristic method based on modularity. But it is difficult for the sequential Louvain method to deal with large-scale graphs. In order to overcome the drawback, researchers have proposed several parallel Louvain methods (Parallel Louvain Method, PLM), which suffer two challenges: (1) latency in the information synchronization and (2) communities swap. To tackle these two challenges, we propose a graph partition algorithm for the parallel Louvain method. Different from existing graph partition algorithms, our graph partition algorithm divides the graph into subgraphs called isolate sets, in which vertices are relatively decoupled from others, and the PLM computes and synchronizes information without delay and communities swap. We first describe concepts and properties of isolate sets. In the second place, we propose an algorithm to divide the graph into isolate sets, which enjoys the same computation complexity as the breadth-first search. Finally, we propose the isolate-set-based parallel Louvain method, which calculates and updates vertices information without latency and communities swap. We implement our method with OpenMP on an 8-cores PC. Experiments on 18 graphs show that our parallel method achieves a maximum 4.62 $\times$ speedup compared with the sequential method, and outputs higher modularity on 14 graphs.

## Introduction

Nowadays, Web technologies, which are bases of various tasks including social networks, financial analyses and so on, have greatly facilitated scientific research and folks’ daily life^1–6^.

An emerging difficulty in Web-technology-based tasks lies in dealing with graph network data, undoubtedly, which are much more complicated than traditional structured data such as lists and matrices.

Community detection, whose aim is to cluster vertices in the graph into communities, is one of the foundational graph algorithms^7–9^.

According to the density of vertices in the graph, community detection divides closely connected vertices into the same communities.

An interesting finding is that the community detection algorithm is an optimization algorithm with “community quality”as the objective function.

According to different objective functions, many kinds of community detection methods are proposed ^10–12^, among which the Louvain method is a heuristic method based on modularity.

Louvain method was proposed by Blondel et al. of Louvain University in 2008^13^.

This method has become increasingly popular owing to its ability to detect high modularity community partitions in a fast and memory efficient manner^14^.

And it has been widely utilized in plenty of applications due to its rapid convergence properties, high modularity, and hierarchical partitioning^15^.

However, with the growth of networks sizes, the scales of graphs are consequently increasing, which means that it may be highly time-consuming for the Louvain method; therefore, many researchers have proposed parallel Louvain algorithms^16,17^.

Every coin has two sides: the introduction of parallelization indeed accelerates Louvain but also brings implementation troubles.

Generally speaking, two major difficulties exist in PLMs: one is the latency in the synchronization of vertices information, and the other is the vertices swap.

To be specific, the first one is the latency in the synchronization of vertices information in the process of parallel computing.

In the serial Louvain method, all vertices in the graph are accessed sequentially, and the calculation and synchronization of vertices are carried out in sequence.

The calculation of each vertex is based on the latest information of the formerly calculated vertex.

In the PLMs, the parallel computing vertices have waiting time for other related vertices to synchronize information.

This latency would delay the convergence of PLMs, and reduce the modularities of graphs;

As for the second difficulty, when two different vertices move to each other’s community after calculation, the vertices swap occurs^18–24^.

After the vertices swap, few mergers between the vertex happens, and the community structure remains.

The exchanged vertices only affect the community labels without improving the modularity of the community.

Therefore, the vertices swap in the computing process will also hurt the convergence of the method.

Most existing PLMs deal with the first difficulty in two ways: one is synchronizing information by compare and swap (CAS) vertices information in hash tables^15^, the other one is using “ghost”vertices (or virtual vertices) between subgraphs in different process^25^. These two existing solutions do not remove the latency entirely and have additional calculation and memory overheads.

And these existing PLMs deal with the second problem by labeling scheme, which reduces the modularity of PLMs.

From the existing PLMs, we find that the above two major difficulties of latency of synchronization and vertices swap are related to the topology of the graph.

In the PLMs, the difficulty of vertices swap is related to the parallel computing of adjacent vertices.

What is more, the latency of information synchronization is not only caused by parallel computing of the adjacent vertices, but also by the vertices with common adjacent vertices.

In order to improve the efficiency of parallel computing without additional overhead, the parallel computing vertices are expected to decouple from their adjacent vertices and the vertices with common adjacent vertices.

In order to solve the bottlenecks of synchronization latency and vertex swap in the parallelization of Louvain method, we propose a novel graph partition algorithm of isolate sets partition algorithm, which divides adjacent vertices and vertices with common adjacent vertices into a series of subgraphs.

Therefore, the vertices in an isolate set are relatively independent in parallel computing, and cannot swap with each other.

Then we propose a PLM based on the isolate sets. It should be noted that our method requires undirected graphs.

We divide the graph network into subgraphs called isolate sets. Using properties of isolate sets, vertices in the same isolate set can be computed and synchronized without latency and without additional overheads. And vertices in the same isolate set are unable to swap communities naturally. Our methods are implemented on an 8-cores personal, and extensive experiments show that the proposed parallel method achieves a speedup of 4.62$\times$ compared to the sequential Louvain method, and meanwhile our method improves the modularity of communities in most cases.

The main contributions of this paper can be summarized as follows:

- The definition and certain properties of the isolate set are proposed. Especially the vertices in isolate sets have properties of relative independence, which is the base of our method.
- An algorithm for dividing graphs into isolate sets is given, and its computation complexity is equivalent to breadth-first search. Our partition algorithm might be not only used in parallel Louvain methods but also implemented on other parallel graph algorithms.
- Isolate-set-based parallel Louvain method (IPLM) is proposed. Experiments show our method is scalable with higher modularity than existing PLMs on the shared memory architecture.

## Results

This section employs 18 frequently used graphs. The experiment mainly shows the modularity and speedup of our method. The experimental results on popular datasets and analyses are reported as below.

### Experimental environment

The hardware platform used in the experiment is a single-node personal PC with i9-9900K and 32G memory. The experiment machine runs the windows server 2019 standard version operation system. Experimental programs all use the cl compiler of Visual Studio 2017 without optimized compilation instructions. Every graph dataset is tested 3 times, and the average is used.

We test two parallel Louvain methods including isolate-set-based PLM and hash-table-based PLM. The sequential method for the test is the sequential Louvain method of C++ program on the personal webpage of Louvain method’s author Vincent D.Blondel^13^. The benchmark of the test is the parallel Louvain methods adapted from the parallel Louvain method based on hash tables^15^.

### Datasets

The graph datasets are from the KONECT project of the University of Koblenz’Landau^26^, the SNAP project of Stanford University^27^, and the datasets of the laboratory for Web Algorithmics^28^. The datasets details are as follows Table 1.

**Table 1:** Details of datasets.

Dataset	Vetices	Edges	Average degree
Gowalla-edges	196,591	950,327	9.6681
Amazon0302	262,111	899,792	6.8658
com-dblp.ungraph	317,081	1,049,867	6.6221
amazon.ungraph	334,863	925,872	5.5299
Amazon0312	400,727	2,349,869	11.7280
Amazon0601	403,394	2,443,408	12.1142
Amazon0505	410,236	2,439,437	11.8928
web-Google	875,713	4,322,051	9.8709
com-youtube.ungraph	1,134,890	2,987,624	5.2650
soc-pokec-relationships	1,632,803	22,301,964	27.3174
as-skitter	1,696,415	11,095,298	13.0808
com-orkut.ungraph	3,072,441	117,185,083	76.2814
cit-Patents	3,774,768	16,518,947	8.7523
com-lj.ungraph	3,997,962	34,681,189	17.3494
enwiki-2013	4,206,785	101,355,853	48.1868
soc-LiveJournal1	4,847,571	43,110,428	17.7864
orkut-groupmemberships	8,730,857	327,036,519	74.9151
wikipedia-link-en	12,082,987	375,535,932	62.1595

### Comparison with PLM of HashTable method

**Figure 1:**
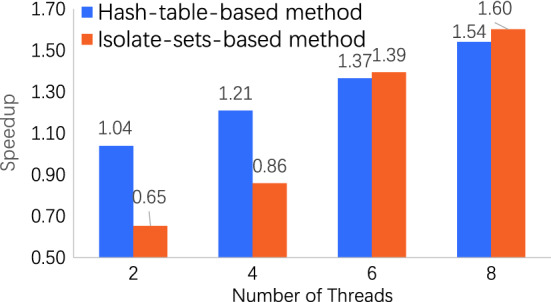
Speedup on dataset LiveJournal. The speedup of our method is higher than hash-table-based method with 8 threads.

**Figure 2:**
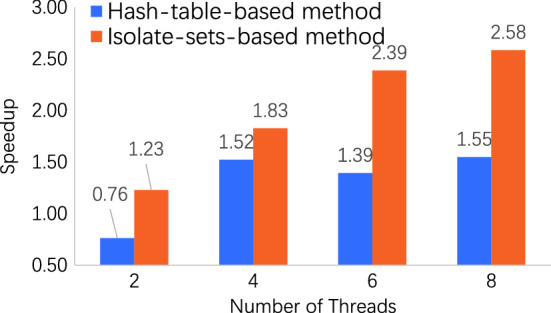
Speedup on dataset wikipedia. Our method has an obviously higher speedup than the hash-table-based method.

In the aspect of information synchronization and communities swap, the benchmark is hash-table-based PLM. We compare with the existing research^15^ that uses hash tables to organize vertices and edges and synchronize information. However, the method of Que et al.^15^ worked on the Power7-based supercomputer Blue Gene/Q (a Power7-IH system and a massively parallel machine). Huge distinctions in the processor architecture and operation system exist between Blue Gene/Q and x86 personal computer. Therefore, we implement both their hash-table-based PLM and our isolate-set-based PLM method in the x86 environment. And we compare the speedup on the same graph datasets of LiveJournal and Wikipedia. The experiment results are shown in the Figs. 1 and 2.

**Table 2:** Long tail of isolate sets.

Dataset	Vetices	Edges	Average degree	Num of isolate sets	Num of isolate sets$^1$	Num of isolate sets$^2$
wikipedia	12,082,987	375,535,932	62.1595	1327	3	13
com-lj	3,997,962	34,681,189	17.3494	450	9	17

^1^Num of isolate sets covering 80% vertices. ^2^Num of isolate sets covering 90% vertices.

**Figure 3:**
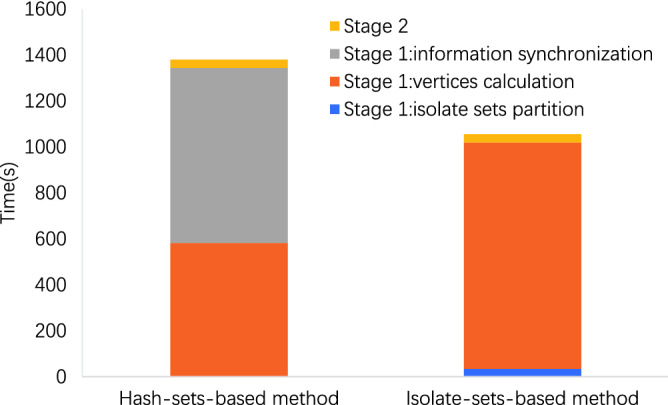
Time breakdown on dataset LiveJournal. The time occupation of isolate sets partition is about 3.3% with 17 iterations, and the time of information synchronization is about 55.2%.

**Figure 4:**
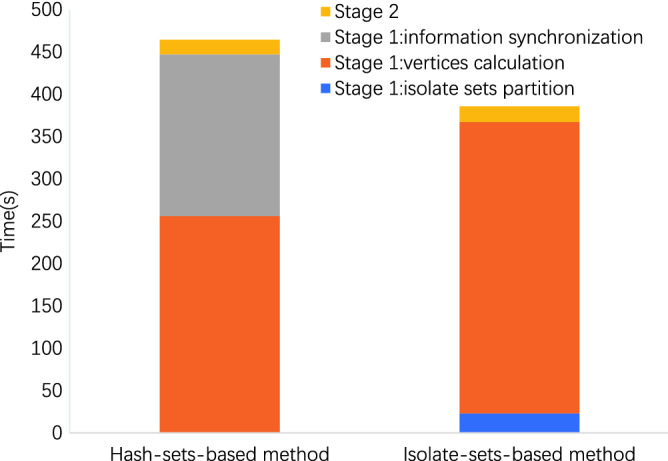
Time breakdown on dataset wikipedia. The time occupation of isolate sets partition is about 6.0% with 13 iterations, and and the time of information synchronization is about 41.2%.

Our method has better performance on the dataset Wikipedia. As shown in Table 2, in the dataset wikipedia, the graph is covered by 1327 isolate sets. While 90% of vertices are included in the first 13 isolate sets (0.98% of all isolate sets), the remaining 1314 isolate sets contain only 10% vertices of the graph. By contrast, the dataset LiveJournal is covered by 450 isolate sets and 90% of vertices are included in the first 17 isolate sets (3.78% of all isolate sets). Obviously, vertices in LiveJournal are connected stronger, which affects the efficiency of our method.

On the dataset LiveJournal, our method has a little lower speedup than hash-table-based method with 2 threads, because this graph has less edges (34,681,189) than others. On this graph, the hash-table-based method has less waiting time for information synchronization, while the isolate-set-based method needs more time on the partitioning graph. However, the increasing threads reduce more computation time on our method than the hash-table-based one. And the speedup of our method is higher than hash-table-based method with 8 threads.

On the dataset Wikipedia, our isolate-set-based method has an obviously higher speedup than the existing method. Because the graph dataset Wikipedia has a large number of edges of 101,355,853. Hash-table-based method requires more waiting time to synchronize and update information. However, the latency in the information synchronization is reduced in our method, which enlarges the speedup of our method dramatically.

As shown in the Figs. 3 and 4, in our method, the time occupation of isolate sets partition is about 3.3% on LiveJournal and 6.0% on Wikipedia, respectively. Our partition algorithm costs less time on partitioning and consequently reduces much time on the information synchronization.

**Figure 5:**
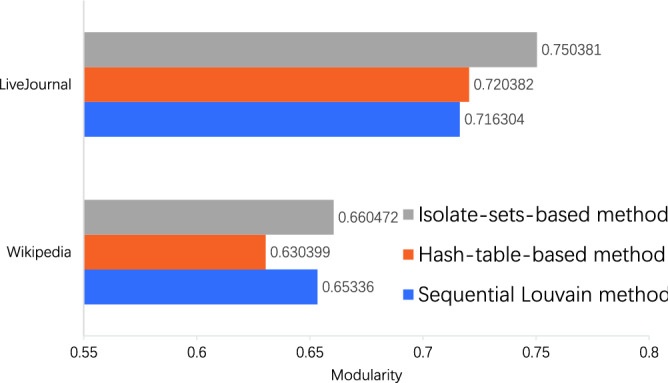
Modularity comparison.

In the perspective of the community detection modularity, our method deals with the communities swap by properties of isolate sets. Restriction vanishes on the movement of vertices in our method, which indicates that the method has more searching spaces. Therefore, our method outputs higher modularity than other methods such as the sequential Louvain method and hash-table-based PLMs. The comparisons of the original Louvain method, hash-table-based method, and our method are shown in the Fig. 5.

### Experiments on extended graph datasets

#### Speedup experiments

**Figure 6:**
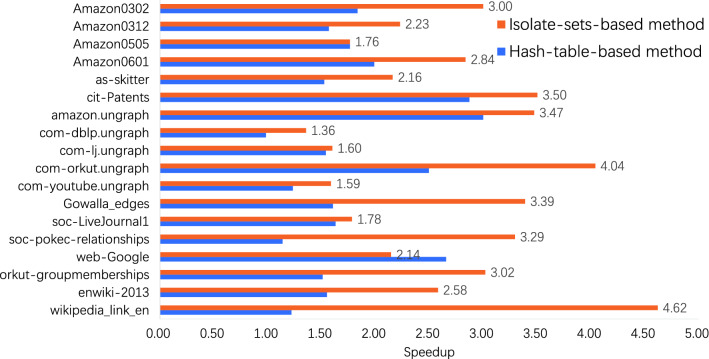
Speedup on extended graph datasets

The improvement on the speedup of our method on extended graph datasets is shown in the Fig. 6.

We implement hash-table-based method and our isolate-set-based method on 18 graph datasets(including the dataset LiveJournal and Wikipedia). These datasets have vertices from 196,591 to 12,082,987, and edges from 899,792 to 375,535,932. Our method presents a higher speedup than hash-table-based method on 17 graph datasets. The exception is the dataset web-Google, on which our method has 2.14$\times$ speedup and is lower than the hash-table-based method of 2.66$\times$. On the dataset Wikipedia-link-en, our method obtains 4.62$\times$ speedup with 8 threads (the best case). In contrast, the speedup is 1.22$\times$ with hash-table-based method. What’s more, the average speedup of our method on these 18 datasets is 2.69$\times$, which is higher than hash-table-based method of 1.78$\times$.

#### Experiments of the modularity

The final community detection performance of our method on extended graph datasets is shown in Table 3.

**Table 3:** Modularity of extended graph datasets.

Datasets	Modularity (NMI)		
	Sequential Louvain method	Hash-table-based method	Isolate-set-based method
Amazon0302	0.8998	0.8969	**0.9017**
Amazon0312	0.8720	0.8677	**0.8759**
Amazon0505	0.8664	0.8713	**0.8765**
Amazon0601	0.8691	0.8721	**0.8776**
as-skitter	0.8132	0.8312	**0.8506**
cit-Patents	0.8114	0.8102	**0.8136**
amazon.ungraph	**0.9262** (0.1240)	0.9248 (0.1182)	0.9261 (0.1240)
com-dblp.ungraph	0.8203 (0.1345)	0.8155 (0.1301)	**0.8211** (0.1345)
com-lj.ungraph	0.7163	0.7204	**0.7504**
com-orkut.ungraph	0.6614 (0.0633)	**0.6987** (0.0645)	0.6567 (0.0627)
com-youtube.ungraph	0.7103	0.6909	**0.7189**
Gowalla-edges	0.6889	0.6839	**0.7115**
soc-LiveJournal1	0.7284	0.7287	**0.7558**
soc-pokec-relationships	0.6895	0.7122	**0.7166**
web-Google	**0.9777**	0.9768	0.9776
orkut-groupmemberships	0.3071	0.3042	**0.3138**
enwiki-2013	0.6534	0.6304	**0.6605**
wikipedia-link-en	0.3618	**0.3817**	0.3706

Significant values are in bold.

As shown in Table 3, our method obtains the highest modularity on the 14 of total of 18 datasets. The sequential Louvain method obtains the highest modularity on 2 datasets. On the datasets amazon.ungraph and web-Google, the modularity of isolate-set-based method is lower than the sequential method by 0.01%. Among the datasets, com-dblp, amazon, and com-orkut have the ground-truths. We compare the NMI of the three methods on these datasets. The results show that there is a slight difference between these three methods.

However, on the datesets as-skitter and com-lj.ungraph, our methods’ improvements are respectively 4.60% and 4.76% than the sequential method. What’s more, our method can improve the quality of community detection in most cases.

## Discussion

To further accelerate Louvain method of community detection method, we propose the definition of isolate sets in a graph, and prove several useful properties of the isolate sets.

Additionally, a graph partition algorithm is proposed, which partitions vertices of a graph into a series of isolate sets.

We propose a parallel Louvain method based on the isolate set partition algorithm. The isolate-set-based PLM takes advantage of the properties of the isolate set to synchronize and update information efficiently in parallel computing without latency.

After the first stage of the Louvain method, two vertices belonging to different communities join the communities that the other vertex is in, which is called community swap.

In the sequential Louvain method, only one vertex is computed and moved at a time. By contrast, several vertices are moved at the same time in PLMs. Existing studies solved the problem by the minimum label heuristic method, which restricts the direction of movement of vertices and decreases the search space. In our method, because of the properties of isolate sets, the vertices and their neighbors fail to fall in the same isolate set. A vertex and its neighbor cannot be moved at the same time. Therefore, the community swap problem is avoided.

Our method requires no extra computation and memory overhead and increases the searching spaces of the method and improves the quality of community detection.

The limitation of our method is that partitioning isolate sets needs extra time. And we will improve the efficiency of the partition algorithm.

To verify the efficiency of our method, we use 18 graph datasets and conduct comparisons with state-of-the-art methods. The experiments results show that our method obtains 4.62 $\times$ speedup on the personal computer with 8 threads and improves the modularity of communities detection in most cases. However, our method degrades a little on a small graph, because the partition algorithm occupies a constant time.

Theoretically, the method depends on the statistical mode of degrees of vertices in the graph, which means the majority of vertices are connected strongly or weakly. When most vertices are connected strongly, the “tail” of the distribution of isolate sets is long, and there are plenty of isolate sets containing few vertices.

In the future, we will improve the efficiency of our method algorithm and implement our method on multicore architecture for community detection on large-scale graphs.

## Methods

We observe that the two challenges mentioned in the last section are related to the topology of the graphs.

The parallel computing vertices are expected to be relatively independent of each other, and the relatively independent vertices reduce the latency in synchronization and thus avoid communities swap.

Therefore, we propose an isolate sets partition algorithm, which divides the graph to isolate sets.

The vertices in the same isolate set are relatively independent, which means that they have no common adjacent vertices. And our partition method has a computation complexity equivalently to the breadth-first search (BFS).

### Definition and properties of isolate sets

In order to help to propose and describe our isolate partition algorithm, we give several definitions and lemmas. We define the dependency set, isolate set^29–32^, and maximum isolate set. Based on the definitions, we prove that the maximum isolate set is non-unique and that an undirected graph can be covered by a series of maximum isolate sets.

#### Definition 1

Given an undirected graph network *G*(*V*, *E*), $\forall v\in G$, ${{N}^{+}}\left( v \right) =\left\{ v \right\} \cup N\left( v \right)$. The set ${{N}^{+}}$ is defined as the dependency set of vertex *v*.

In a graph *G*(*V*, *E*), the union of a vertex *v* and the neighborhoods of *v*( *N*(*v*)) is defined as the dependency set of vertex *v*. Obviously, the dependency set of vertex *v* consists of vertex *v* and all adjacent vertices of vertex *v*.

#### Definition 2

In a graph network *G*(*V*, *E*), $s\subseteq V$ is called an isolate set, if $\forall {{v}_{i}},{{v}_{j}}\in s$ and ${{v}_{i}}\ne {{v}_{j}}$, ${{N}^{+}}\left( {{v}_{i}} \right) \cap {{N}^{+}}\left( {{v}_{j}} \right) =\emptyset$.

In a graph network *G*(*V*, *E*), the set *s* is a subset of *V*. Vertices $v_{i}$ and $v_{j}$ are arbitrary two vertices in the set *s*, and sets ${{N}^{+}}\left( {{v}_{i}} \right)$ and ${{N}^{+}}\left( {{v}_{j}} \right)$ are the dependency sets of $v_{i}$ and $v_{j}$. If the intersection of these two dependency sets is empty set, the subset *s* is called an isolate set of graph *G*.

According to Definition 2, intersections of the dependency sets of vertices in an isolate set are empty.

Sometimes, the isolate set is similar to the independent set, in which the vertices have no common edges. What distinguishes is that two arbitrary vertices in an isolate set have no common adjacent vertices. Especially, the sets of the single vertex are isolate sets.

#### Definition 3

Given an undirected graph network *G*(*V*, *E*), *s* is an isolate set of *G*. $\forall {{v}_{j}}\in G-s$, $\exists {{v}_{i}}\in s$, ${{N}^{+}}\left( {{v}_{i}} \right) \cap {{N}^{+}}\left( {{v}_{j}} \right) \ne \emptyset$, the isolate set *s* is defined as an maximum isolate set of graph *G*.

In a graph *G*(*V*, *E*), *s* is an isolate set of *G*. Two arbitrary vertices ${{v}_{j}}\in G-s$ and the vertex ${{v}_{i}}\in s$ exist, which cannot make the intersection of these two dependency sets ${{N}^{+}}\left( {{v}_{i}} \right)$ and ${{N}^{+}}\left( {{v}_{j}} \right)$ empty set. The isolate set *s* is an maximum isolate set of the graph *G*. That is to say, there is no vertex in set $G-s$ that can be divided to the isolate set *s*.

**Figure 7:**
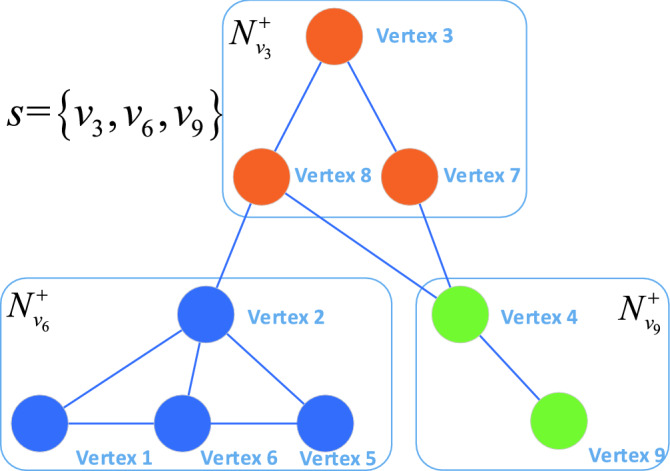
Isolate set. One of the isolate sets in the example is set *s*, which contains vertices $v_3$, $v_6$ and $v_9$. The sets ${N}^{+}_{v_3}$, ${N}^{+}_{v_6}$ and ${N}^{+}_{v_9}$ are dependency sets of vertices $v_3$, $v_6$ and $v_9$.

#### Lemma 1

The maximum isolate set is non-unique. Except for the graph *G*(*V*, *E*) including one vertex, the maximum isolate set of *G* is unique, and the isolate set of *G* is *V*.

#### Proof 1

Suppose there is a graph *G* including 2 vertices at least, and the isolate set *s* is the only maximum isolate set of *G*. There is an arbitrary vertex *v* located in the complementary set of graph *G* ($\forall v\in G-s$). The vertex *v* forms a set $s^{*}$ containing itself ($s^{*}=\left\{ v \right\}$). According to the Definition 2, the set $s^{*}$ is another isolate set of graph *G*. Hence, if the set $s^{*}$ is a maximum isolate set, it conflicts with the former assumption that the maximum isolate set is unique. If the set $s^{*}$ is not a maximum isolate set, there must be a maximum isolate set including the set $s^{*}$, which is different from the set *s* and also conflicts with the assumption. $\square$

#### Lemma 2

Given an undirected graph *G*(*V*, *E*), $\exists {{s}_{i}}\subset G$ ( ${s}_{i}$ is isolate), $\bigcup \nolimits _{i}^{n}{{{s}_{i}}}=G(V,E)$.

#### Proof 2

Given an undirected graph *G*(*V*, *E*), we can find a series of isolate sets $\{ s_{i}\}$. The graph *G* can be covered with union of these isolate sets.

Suppose the graph *G* cannot be covered with the union of isolate sets $\bigcup \nolimits _{i}^{n}{{{s}_{i}}}$, and a vertex ${{v}_{0}}\in G-\bigcup \nolimits _{i}^{n}{{{s}_{i}}}$.

According to the Lemma 1, we divide the vertex into a new isolate set ${{s}_{n+1}}=\left\{ {{v}_{0}} \right\}$. The union of isolate set $\bigcup \nolimits _{i}^{n+1}{{{s}_{i}}}$ covers the graph *G*, which conflicts with the former assumption. Therefore, we can find a series of isolate sets $s_{i}$ to cover the graph *G*. $\square$

#### Theorem 1

For $\forall {{v}_{i}}\in G(V,E)$ and $\forall {{v}_{j}}\in {{N}^{+}}\left( {{v}_{i}} \right)$, there exists a set ${{B}_{i}}={{N}^{+}}({{v}_{i}})\cup \bigcup \nolimits _{j}{{{N}^{+}}({{v}_{j}})}$, $\forall {{v}_{k}}\in G-{{B}_{i}}$, where $v_{i}$ and $v_{k}$ are in the same isolate set.

In an undirected graph *G*(*V*, *E*), an arbitrary vertex $v_{i}$ and the neighborhood vertices $v_j$ are included in the set ${{N}^{+}}\left( {{v}_{i}} \right)$. The adjacent vertices of $v_{j}$ are included in the set ${{N}^{+}}\left( {{v}_{j}} \right)$. The union of set ${{N}^{+}}\left( {{v}_{i}} \right)$ and sets ${{N}^{+}}\left( {{v}_{j}} \right)$ admits set $B_{i}$. And an arbitrary vertex $v_{k}$ in the complementary set of *G* ($v_k\in G-B_{i}$) is in the same isolate set as the vertex $v_{i}$.

#### Proof 3

In an undirected graph *G*(*V*, *E*), vertex $v_{i}$ is an arbitrary vertex in isolate set $s_{i}$. According to the Definition 2, vertices $v_{j}$ ($v_{j}\in N^{+}(v_i)$, the neighborhood vertices of $v_{i}$) are not in the isolate set $s_{i}$. And the adjacent vertices of $v_{j}$ (vertices in $N^{+}(v_j)$) are not in the isolate set $s_{i}$, either due to that these vertices and the vertex $v_{i}$ have common adjacent vertices of $v_{j}$. The set $B_i$ is union of the set ${{N}^{+}}\left( {{v}_{i}} \right)$ and the set ${{N}^{+}}\left( {{v}_{j}} \right)$. Then, all the vertices are in the set $B_{i}$, in which the vertices either are adjacent vertices of $v_{i}$ or have common adjacent vertices with $v_{i}$. Therefore, an arbitrary vertex in complementary set of *G* ($G-B_{i}$) is in the same isolate set $s_{i}$ with the vertex $v_{i}$. $\square$

The Lemmas 1 and 2 indicate that the maximum isolate sets can be found by the graph search algorithm. And we propose an algorithm for partitioning the graph into maximum isolate sets.

### Isolate sets partition algorithm

According to the definition of isolate sets, in an isolate set the neighborhood of a vertex are totally different from the neighborhood of another vertex, which means that these vertices have been decoupled. The decoupling vertices can be parallelly computed without waiting for information synchronization, and the vertices cannot swap with each other in the movement. Therefore, the latency in the information synchronization is reduced without computing and memory overhead. At the same time, the communities swap is avoided.

According to the Lemma 2, an undirected graph can be divided into several isolate sets. The union of these isolate sets contains the whole vertices. In the first stage of the Louvain method, isolate sets are computed serially, and then vertices in the same isolate set are calculated in parallel to accelerate the Louvain method. However, the intersection of these isolate sets may not be empty. Such a fact means these isolate sets may share the same vertices, which are computed more than one time, unreasonably.

This part proposes a novel graph partition algorithm based on breadth-first search. Our algorithm enjoys the advantage of the avoidance of the repeat computation for the intersections. The algorithm divides the whole vertices of an undirected graph to several isolate sets. The union of these isolate sets covers vertices *V* of the graph *G*, and the intersections of these sets are empty, that is, we find ${{s}_{1}},{{s}_{2}},{{s}_{3}},\cdots ,{{s}_{n}}\subset G\left( V,E \right)$ such that $\bigcup \nolimits _{i}{{{s}_{i}}}=V$ and $\bigcap \nolimits _{i}{{s_i}} = \emptyset$. The pseudocodes are displayed in Algorithm 1. 
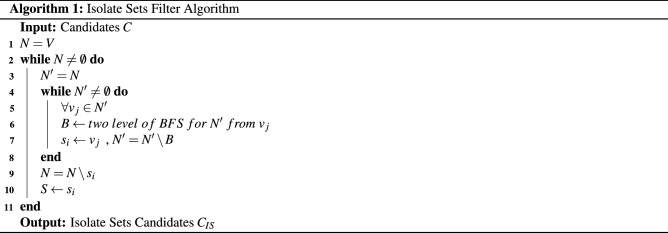


Our algorithm contains 3 steps:

In the first step, the set *N* is initialized with vertices set *V*. In other words, the set *N* includes all vertices in the graph.

In the second step, we search an isolate set $s_i$ from the set *N*.

Firstly, a set $N'$ is initialized with the set *N*.

Secondly, the two-level breadth-first search is implemented from an arbitrary vertex $v_{j}$ in the set $N'$. After searching, these searched vertices form the set *B*. That is to say, the set *B* is the union of adjacent vertices of $v_{j}$ and the neighbor vertices of adjacent vertices of $v_{j}$.

Thirdly, the vertex $v_{j}$ is moved into the isolate set $s_{i}$, and the other vertices in the set *B* are moved out of the set $N'$. These three processes are implemented repeatedly until the set $N'$ is an empty set.

When the set $N'$ becomes an empty set, the isolate set $s_{i}$ is found out.

In the third step, the vertices in the isolate sets are moved out of the set *N*. And the same operation as step 2 and step 3 is implemented alternatively until the set *N* becomes an empty set. When the set *N* is empty, we get the desired non-intersected isolate sets whose union keeps all vertices.

What surprised is that our algorithm enjoys quite low computation complexity from the experimental observation. Although it is quite hard to prove such a phenomenon rigorously, several explanations may help the understanding: Let *m* be number of the edges and *n* be the number of vertices, respectively. In the process of generating an isolate set, all the edges in the graph are traversed by breadth-first search only once. And the vertices of the graph are traversed by breadth-first search once. The computation complexity of this part is equal to the breadth-first search, which is $O(n+m)$. Then the traversed vertices are moved once, which has the computation complexity of *O*(*n*). The computation complexity of generating an isolate set is $O(2n+m)$. These steps are implemented iteratively, the total computation complexity of our partition algorithm is $O(k(2n+m))$, where *k* is the iteration number of the algorithm. In the experiments, we found that *k* is always small. Therefore, the isolate set partition algorithm does not degrade to the breadth-first search algorithm too much in the perspective of computation complexity. How to bound *k* theoretically will be left as future work.

### Isolate-set-based parallel louvain method

According to the timing of information synchronization, prior works on synchronization latency indicate two types: non-real-time synchronization and real-time synchronization.

The non-real-time synchronization method utilizes the former information to implement the calculation and does not synchronize vertices information immediately after vertices calculation.

Many PLMs with a non-real-time method have been proposed, among which different synchronization opportunities are selected. Therefore, the latencies of these methods are different.

In some studies, the vertices information is updated at the end of stage 1 of the Louvain method, and the latency is the entire iteration time.

The speedup of these methods is from 3 to 6$\times$ based on OpenMP^14^ and 12$\times$ based on GPUs^33^.

To reduce the delay of synchronization, in some studies^25,34–38^, the graph is partitioned into subgraphs. During an iteration, information is updated when a subgraph has finished computing.

The real-time synchronization method was proposed by Que et al.^15^. They utilize thread-safe hash tables to manage the vertices’information.

Parallel computing vertices in different threads read and update information in the hash table in parallel by Compare and Swap (CAS) operations, which guarantees the updated information is accessed to the other vertices (threads).

This work achieves a speedup of 49.8$\times$ in the“Giant Blue” supercomputer.

Naim et al.^39^ use two hash tables to manage the vertices information and communities information, which is different from the previous studies.

The authors implement their method on GPUs, and their work ultimately reaches the highest speedup of 270$\times$ on several medium graphs.

However, the hash tables have massive storage overhead and computation overhead, which is unaffordable for personal computers.

Moreover, the hash-table-based methods cannot eliminate the latency entirely, and it is difficult for hash-table-based methods to deal with large complex graphs on personal computers, which usually have less memory than supercomputers.

#### Information synchronization and vertices movement

In the first stage of Louvain method, every vertex is traversed. When traversed, the vertex utilizes information about itself and its neighbors for the calculation to update. Unfortunately, latency always exists in the updating, which limits the power of parallelism. Among the current PLMs, one popular methodology uses the hash tables, in which vertices’ information is organized by hash tables. When different vertices utilize and update the information of the same vertex at the same time, the CAS operation (compare-and-swap is an atomic instruction) guarantees the correct operation sequence of writing first and then reading. The computed information can be used to update other vertices. In this way, the vertices are computed parallelly.

Nevertheless, two bottlenecks trouble hash tables synchronizing information. The first one is that hash tables need additional memory and computation overhead. On a graph with hundreds of millions of vertices, methods based on hash tables require huge memory, which may overdrive personal computers’ memory. The second one is that the likelihood of collision increases. When several parallel computing vertices modify the information of the same location, a significant waiting time is needed for completing these operations.

What’s more, state-of-the-art PLMs deal with the communities swap by minimum label heuristic method. The minimum label heuristic method labels communities and utilize the notation to guide vertices movements, which restricts vertices movements and decreases the modularity of community detection.

To this end, we propose a parallel Louvain method based on isolate sets. Vertices in the same isolate set are decoupled. In our method, the parallel computing vertices are in the same isolate set. That is to say, these vertices have completely different neighbors. The parallel computing vertices in an isolate set can synchronize information in time without memory and computation overhead. In the first stage of the Louvain method, the computation of these vertices utilize information of their neighbor vertices without waiting for other vertices synchronization. After computation, these vertices update their information, which has no influence on other parallel computing vertices. Because of the properties of isolate sets, the vertices and their neighbors fail to fall in the same isolate set. A vertex and its neighbor cannot be moved at the same time. Therefore, the communities swap is avoided.

An example is shown in the Fig. 7. Vertices 3, 6, and 9 belong to isolate set $s_{1}$, which are computed parallelly. The computation of vertex 3 requires the information of vertices 7 and 8. The computation of vertex 6 requires the information of vertices 1, 2, and 5. The computation of vertex 9 requires the information of vertex 4. The parallel computations of vertices 3, 6, and 9 are independent of each other. After computing, these three vertices synchronize information to their neighbor without waiting, because the neighbor vertices are occupied by these three vertices exclusively. And the movement target of vertex 3, 6, and 9 is one of its neighbor vertices, which are not in the parallel computing vertices. The communities swap is avoided.

We implement our method in OpenMP and C++ program language. Due to the shared memory programming of OpenMP, vertices in an isolate set are forked to different threads, and these threads carry out calculations and information synchronization independently. 
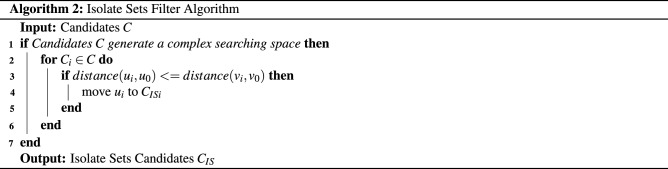


#### Implementation of the algorithm

From the definition, traversing these isolate sets is equal to traversing all the vertices. The existing PLMs traverse the vertices in order of their index. Different from the existing Louvain methods, our method traverses the vertices in order of the isolate sets. Our method computes vertices in the same isolate set parallelly. During the parallel computing of vertices, the information is synchronized in time, and the vertices are moved parallelly. In this way, the method utilizes the property of isolate sets to automatically synchronize information in an isolate set and update community information without relying on other information synchronization methods, such as hash tables. Therefore, all vertices in the graph are computed without latency and communities swap in the first stage of Louvain method.

The isolate-set-based parallel Louvain method involves three stages and the pseudocodes are displayed in Algorithm 1:

The first step is to partition the graph into a series of isolate sets. These isolate sets can cover all the vertices in the graph, and the intersection of these isolate sets is an empty set.

The second step is to iteratively traverse these isolate sets and calculate the community information of the vertices. The first time of the second stage of the method being executed, the communities’ information of all vertices needs to be initialized, and each vertex in the graph is divided into a different community. After the initialization is completed, these isolate sets are traversed in turn. When the isolate set is traversed, the vertices in this isolate set are computed parallelly. After computation, these vertices are moved in parallel to the communities of their largest $\Delta Q$, and synchronize their information. Then this stage is implemented iteratively until the modularity of the entire graph no longer changes.

The third step is to restructure the graph, which merge vertices belonging to the same community into a new vertex, according to the updated vertices information. After the second stage of calculation, the vertices in the graph are moved to the neighbor vertices of the largest increase in modularity. And the communities information of vertices has been changed. The vertices belonging to the same community are merged into a new vertex, and the edges between these vertices are ignored. The edges between different communities are reserved.

These three steps are applied alternatively on the reconstructed graph until the modularity no longer changes or the change of modularity is less than a certain threshold. The threshold $t_{threshold}$ is a small quantity, which improves the robustness of the algorithm and ensures that the algorithm ends properly. What’s more, the threshold avoids the extreme cases where all communities are merged into one community. And the final community detection result is obtained.

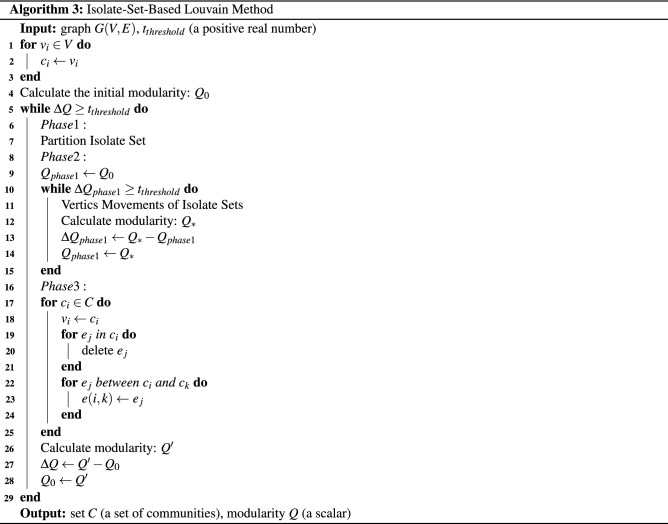



## Data Availability

The datasets analysed during the current study are available in the SNAP repository, https://snap.stanford.edu/data/index.html.
